# Plant structural complexity and mechanical defenses mediate predator–prey interactions in an odonate–bird system

**DOI:** 10.1002/ece3.2705

**Published:** 2017-02-10

**Authors:** Patrick Grof‐Tisza, Eric LoPresti, Sacha K. Heath, Richard Karban

**Affiliations:** ^1^ Department of Entomology and NematologyUniversity of CaliforniaDavisCAUSA; ^2^ Ecology Graduate GroupUniversity of CaliforniaDavisCAUSA; ^3^ Department of Environmental Science and PolicyUniversity of CaliforniaDavisCAUSA

**Keywords:** associational refuge, indirect effects of species interactions, positive facilitation, predation refuge, Red‐winged Blackbirds

## Abstract

Habitat‐forming species provide refuges for a variety of associating species; these refuges may mediate interactions between species differently depending on the functional traits of the habitat‐forming species. We investigated refuge provisioning by plants with different functional traits for dragonfly and damselfly (Odonata: Anisoptera and Zygoptera) nymphs emerging from water bodies to molt into their adult stage. During this period, nymphs experience high levels of predation by birds. On the shores of a small pond, plants with mechanical defenses (e.g., thorns and prickles) and high structural complexity had higher abundances of odonate exuviae than nearby plants which lacked mechanical defenses and exhibited low structural complexity. To disentangle the relative effects of these two potentially important functional traits on nymph emergence‐site preference and survival, we conducted two fully crossed factorial field experiments using artificial plants. Nymphs showed a strong preference for artificial plants with high structural complexity and to a lesser extent, mechanical defenses. Both functional traits increased nymph survival but through different mechanisms. We suggest that future investigations attempt to experimentally separate the elements contributing to structural complexity to elucidate the mechanistic underpinnings of refuge provisioning.

## 1 Introduction

Refuges are important habitat features that allow prey populations to persist in the presence of their predators (McNair, 1986; Sih, 1987; Werner, Gilliam, Hall, & Mittelbach, 1983). Associational refuges occur when a habitat‐forming species facilitates a second species by provisioning a refuge from abiotic or biotic stress (Bertness, Leonard, Levine, Schmidt, & Ingraham, 1999; Bruno, Stachowicz, & Bertness, 2003). Much research has demonstrated that associational refuges created by plant architecture mediate predator–prey dynamics: animals associated with structurally complex plants often experience reduced predation risk (e.g., Bruno et al., 2003; Grutters, Pollux, Verberk, & Bakker, 2015; Klecka & Boukal, 2014; Rantala, Ilmonen, Koskimaki, Suhonen, & Tynkkynen, 2004; Valinoti, Ho, & Armitage, 2011; Warfe & Barmuta, 2006).

The term “structural complexity” has proved challenging to define and quantify (McCoy & Bell, 1991). Generally, however, it refers to the physical arrangement of objects in space and is quantified by measuring the density of a particular structural element or the heterogeneity of the diversity of those elements (Humphries, La Peyre, & Decossas, 2011; Stoner & Lewis, 1985). Increased structural complexity decreases prey visibility and encounter rates or prevents access by predators if the interstitial space between structural elements is sufficiently small relative to predator body size (Bartholomew, 2002; Crowder & Cooper, 1982; Rilov, Figueira, Lyman, & Crowder, 2007). Consequently, the effectiveness of an associational refuge for an associating organism is in part a function of the degree of structural complexity in the habitat‐forming species. For example, southern pygmy perch caught more prey in the presence of plants with long, flat leaves and fewer in the presence of dense whorls of finely dissected leaves (Warfe & Barmuta, 2004), and eggs of the tansy leaf beetle (*Galeruca tanaceti*) experienced lower parasitism rates when oviposited in plants with a high degree of branching (Obermaier, Heisswolf, Poethke, Randlkofer, & Meiners, 2008). In these two examples, plants with greater structural complexity provided more or higher quality refuges, thereby decreasing the effectiveness of these predators.

Despite the recognition that heterogeneity of structural elements contributes to overall structural complexity, it is often only assessed qualitatively, with a focus on the *number* of different comprising elements as opposed trying to understand their specific effects (Tokeshi & Arakaki, 2012 but see Beck, 2000; Jenkins & Sutherland, 1997; Loke & Todd, 2016). For example, leaves and stems are the two primary structural elements comprising plant canopies. Simply keeping track of the numbers of elements ignores their functional difference in regard to refuge provisioning; stems are rigid structures that may exclude large‐bodied predators, while leaves are non‐ridged structures that provide substantially more cover than stems. Mechanical defenses such as ridged spinose plant structures (e.g., thorns, spines, and prickles; Simpson, 2010) are structural elements of some plant species that have been shown to confer a survival advantage for directly associating species (Grof‐Tisza, Antell, Holyoak, & Karban, 2015). Similarly, sea urchin spines (Townsend & Bologna, 2007) and sea star thorns (Stier, Steele, & Brooks, 2008) can deter predators from accessing refuge‐benefiting individuals. Though mechanical defenses are structural elements, they may affect predator–prey interactions in different mechanistic ways than structural complexity alone. Structural complexity impacts detection rates and access to the prey by the predator; though predator access may be impacted by the presence of mechanical defenses, it is likely through avoidance behavior (i.e., the predator will avoid the mechanical defense that may cause physical injury) above and beyond the access limitation. For this reason, we sought to parse out the relative contribution of structural complexity in terms of the density of individual structural elements affecting prey detection and mechanical defenses in refuge provisioning. Consequently, for the purpose of this study, we consider mechanical defenses separately from structural complexity.

**Figure 1:**
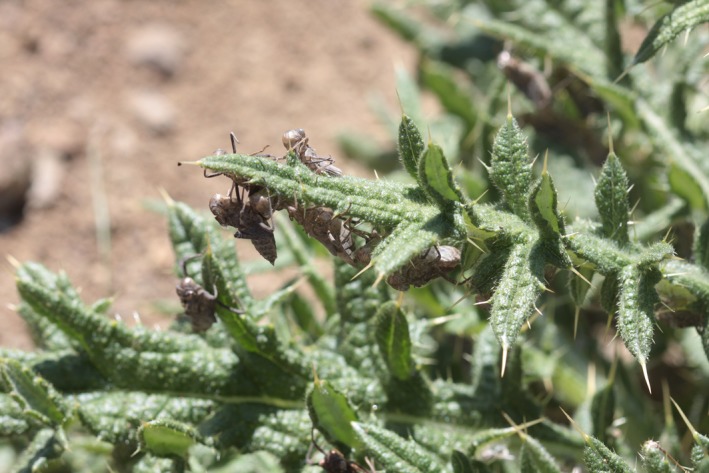
Exuviae of the variegated meadowhawk dragonfly (*Sympetrum corruptum*) on bull thistle (*Cirsium vulgare*). Photograph credit: Eric LoPresti

Dragonfly and damselfly (Odonata: Anisoptera and Zygoptera) nymphs have been used extensively to test hypotheses regarding the influence of structural complexity on predator–prey dynamics and trophic structure (Grutters et al., 2015; Jordan & McCreary, 1996; Warfe & Barmuta, 2004). Though the duration of the larval stage is species and environment‐dependent, all odonates must eventually leave their natal aquatic habitat and choose terrestrial substrates, often plants, on which to undergo their final molt (hereafter, emergence). Apart from nocturnally emerging species, most odonates are vulnerable to predation by insectivorous birds during the site selection and emergence process; they must spend several minutes molting and then several more minutes pumping hemolymph into their wings prior to achieving flight. In some systems, 50% of odonate mortality occurs during this brief window, which generally lasts between 15 and 60 min (Corbet & Brooks, 2008). We observed that on the shores of a small pond at the UC‐Davis McLaughlin Reserve, Lake County, California, USA, odonate exuviae were strikingly more abundant on mechanically defended, structurally complex plants compared to those that were undefended with low structural complexity (Figure 1). We hypothesized that emerging nymphs in structurally complex and mechanically defended plants are more likely to evade predation compared to those in plants that do not possess these physical traits, with structural complexity providing a greater benefit than mechanical defenses alone.

In this study, we first quantified our observations of the natural distribution of odonate exuviae and plant traits surrounding a pond. Second, we conducted two multifactorial field experiments using artificial plants to test the importance of plant structural complexity and mechanical defenses on odonate emergence‐site preference and subsequent survival. Using artificial plants is a common experimental strategy in structural complexity studies (Grutters et al., 2015; Hansen, Sagerman, & Wilkström, 2010; Jeffries, 1993; Rooke, 1986; Taniguchi, Nakano, & Tokeshi, 2003; Warfe & Barmuta, 2004, 2006). We used camera traps during experiments to confirm our assumption that birds were the dominant predators of emerging nymphs and to validate our methodological choice of using odonate exuviae as a proxy for a successful emergence event. The effects of structural complexity on many population and community attributes have been extensively studied (Kovalenko, Thomaz, & Warfe, 2012; Tokeshi & Arakaki, 2012). Comparatively, mechanical defenses, specifically their role in refuge provisioning in terrestrial systems, have received less attention (but see Grof‐Tisza et al., 2015). To our knowledge, this is the first study to investigate the interaction of structural complexity and mechanical defenses in plants on a predator–prey interaction.

## 2 Materials and Methods

### 2.1 Site description

This study took place in a permanent fishless pond in Lake County, California (38.865113N, −122.448036W). A seasonally flowing seep drained into the less than 1‐hectare pond created by an earthen dam at the eastern shore. The dominant shoreline plants include *Centaurea solstitialis*,* Atriplex rosea*,* Trichostema laxum*,* Cirsium vulgare*,* Gnaphalium* spp., *Schoenoplectus californicus*, and *Typha angustifolia*. Roughly, 20 m back from the shoreline is serpentine chaparral, dominated by *Arctostaphylos viscida* and *Quercus durata*. Odonates naturally occur in nearby seeps and ponds, and nearly all species found in the vicinity, except local stream specialists (e.g., *Ophiogomphus* spp.), occur as adults at the studied pond and likely breed there.

### 2.2 Exuviae surveys

To determine how two plant traits (mechanical defenses and structural complexity) correlate with odonate exuviae on vegetation, we removed and counted exuviae from all plants within two, 50 × 2 m plots located 10 m from the edge of our focal pond along the North and South shores. For each plant, we calculated its complexity using a modified index developed by Bartholomew, Diaz, and Cicchetti (2000) and categorized it as possessing or not possessing mechanical defenses, specifically, ridged spinose plant structures (i.e., thorns, spines, or prickles; Simpson, 2010). The cover provided by an individual plant was estimated by multiplying the total canopy volume by its percent canopy cover (percentage of the canopy occupied by foliage and stems). This canopy cover value was then divided by the total canopy volume to create a dimensionless measure of the amount of cover within a plant's canopy. This generates and index on a scale of 0–1 with 1 being the most structurally complex. A plant with a complexity index rating of 1 would have a canopy completely filled with foliage and stems (i.e., no gaps), while a plant that consisted of only stems and no foliage would be at the low end of the scale (A in Appendix S2). Though more rigorous methods to quantify structural complexity are described in the literature (e.g., Bartholomew et al., 2000; Halley et al., 2004), they generally involve extensive measurements and destructive sampling. Our metric is very similar to the estimation of percent plant cover in quadrat sampling but in three‐dimensional space. Consequently, our simple method provides a rapid and reliable means to estimate structural complexity of many plants in a field setting.

We used generalized linear mixed models (glmm) to explore the relationship between plant traits (mechanical defenses and structural complexity) and number of exuviae attached to vegetation within the two plots. The function “glmmADMB” was used to fit models with a negative binomial error distribution and log link function (Fournier et al., 2012; R Core Team 2014; Skaug, Fournier, Nielsen, Magnusson, & Bolker, 2013). Using a stepwise deletion approach from the maximal model including interactions, the minimal adequate model was selected. The minimal adequate model consisted of only significant terms (*p* < .05) assessed by residual deviances to a chi‐square distribution with residual degrees of freedom (Crawley, 2007). Complexity (see derivation of complexity index above) and mechanical defense (presence or absence) were included as fixed effects, and plot and species identity were included as random effects.

### 2.3 Molting preference and predation experiment

**Figure 2:**
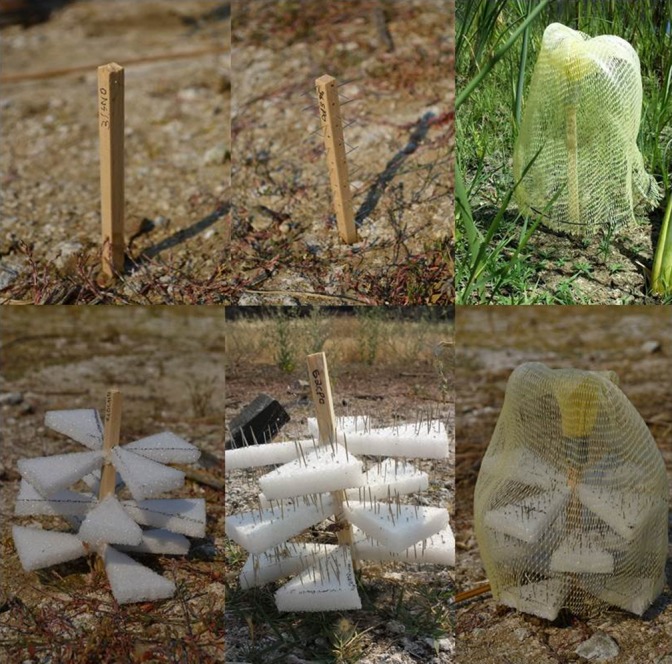
Artificial plants used in the factorial field experiments. Top row from the left: Low structural complexity, no defenses, predator access; low structural complexity, mechanical defenses, predator access; low structural complexity, mechanical defenses, predator exclusion. Bottom row from the left: High structural complexity, no defenses, predator access; High structural complexity, defenses, predator access; high structural complexity, mechanical defenses, predator exclusion. Not shown: Low structural complexity, no defenses, predator exclusion; high structural complexity, no defenses, predator exclusion. Photograph credit: Patrick Grof‐Tisza, Eric Lopresti, Sacha Heath

We conducted a fully crossed factorial field experiment with three factors each with two levels using artificial plants to separate the importance of mechanical defenses and structural complexity on premolting nymph preference as well as predation avoidance. The treatments included the presence or absence of mechanical defenses (*D*), high or low structural complexity (*C*), and the exclusion or access of predators (*P*). The predator‐exclusion treatment allowed us to measure nymph preference for each trait. The number of exuviae on the plants within the predator‐access treatment is a function of how many nymphs initially chose to molt on a particular plant minus how many have been removed by predators. Thus, the predator‐access treatment allowed us to measure the combined effects of preference and survival. Significant interactions between mechanical defenses and structural complexity with the predator‐access/exclusion treatment (i.e., *D* × *P*,* C* × *P*, or *C* × *D* × *P*) would indicate that these functional traits confer a survival advantage. All artificial plants consisted of a square wooden dowel stem (10 mm × 30 cm; Figure 2). The high complexity treatment had three whorls of four styrofoam isosceles‐triangular leaves (*W* × *L*; 6.4 × 5 cm) attached at equal intervals along the stem, with the lowest whorl of leaves at least 3 cm above the ground. The low and high structural complexity treatments had complexity ratings of 0.1 and 0.6 using our complexity index, respectively. The mechanical defense treatment had sewing pins pushed through both the stems and leaves such that they protruded on the opposite side, simulating thistle prickles at a biologically relevant density and length (B in Appendix S2). Predator‐excluded plants had a cage consisting of a wire frame covered in fine mesh suspended over the artificial plant such that a 3‐cm gap was present between the cage and ground. This gap allowed nymphs access to the stem but excluded birds from nymphs above 3 cm. Each treatment combination was replicated 17 times for a total of 136 plants. We haphazardly placed artificial plants within 5 m from water line among naturally occurring vegetation, approximately 30 cm apart on the East and South shores of the focal pond (C in Appendix S2). Because the West and North shores were difficult to access, they were not used in this study. To our knowledge, the bottom of the pond is uniform such that there are no underwater features that correlate with the locations of our plants or with odonate emergence in general. Weekly from 24 June to 24 August 2014, we removed and counted exuviae from the artificial plants. We used the presence of exuviae as a proxy for successful (i.e., nonpredated) emergence events (see Section 5 in results). Our need to remove exuviae on a weekly basis constrained our high structural complexity treatment level. Structural complexity above 0.6 would have increased our handling time considerably and likely resulted in broken leaves. However, our high complexity treatment fell above the 75th percentile of the structural complexity calculated for the naturally occurring plants in our survey plots (see Section 2.2).

Again, we used generalized linear mixed models (glmm) to determine the importance of structural complexity, mechanical defenses, and predation, on the number of exuviae on the artificial plants. Upon analysis (Table 1), we found that nymphs showed a preference for certain plant traits, especially structural complexity. Moreover, an additional predator‐free, exclusion control experiment suggested that nymphs may slightly prefer the exclusion cages (D in Appendix S2). If nymphs did perceive and prefer exclusion cages, the assumption that the number of exuviae in the predator‐access treatment subtracted from the predator‐exclusion treatment represented the number of molting nymphs removed by predators is invalid. This is because the number of exuviae on the predator‐exclusion treatment is a function of nymph preference for both the leaves of the artificial plant and the exclusion cage itself. Consequently, we could not determine the influence of plant traits on nymph survival using higher order interactive effects (i.e., *D* × *P*,* C* × *P*, or *C* × *D* × *P*). Instead, we (1) restricted our analysis for determining nymph preference to the predator‐exclusion treatment, (2) calculated inferred survival using aggregate exuviae values (see below), and (3) conducted a second experiment (see Section 3.3) where we controlled the starting number of nymphs on the artificial plants and quantified the rate of removal by avian predators.

**Table 1:** Aggregate exuviae count totals for artificial plants with (+) and without (−) mechanical defenses and with high or low structural complexity in both the predator‐exclusion and predator‐access treatments. Predator‐exclusion values were adjusted to account for effect of cage which potentially inflated exuviae counts

Mechanical defense	Structural complexity	Exuviae counts	
		Predator exclusion (original, adjusted)	Predator access
−	High	31, 25^a^	25
−	Low	17, 13	7
+	Low	19, 8	8
+	High	66, 51	41

a The actual corrected value is 23 but we limited it to 25 to prevent “zero” values in the analysis.

To estimate preference, we used generalized linear mixed models (glmm) with mechanical and structural complexity as fixed effects and “shore location” and “day” as random effects using exuviae counts from the predator‐exclusion treatment as the dependent variable. To estimate inferred survival, we used aggregated data (i.e., season totals): For each of the complexity and mechanical defense treatments, we subtracted the total number of exuviae from the predator‐access treatment (i.e., preference – depredated) from the total number of exuviae from the exclusion treatment (i.e., preference). If there was no effect of cage on nymph preference, this value would represent the number of depredated molting nymphs for that treatment. However, because we detected a weak effect of cage in some cases, (D in Appendix S2), we used the parameter estimate for the overall effect of cage to adjust the total number of exuviae in all exclusion treatments to account for the potentially inflated exuviae numbers (i.e., we reduced the number of exuviae in the predator‐exclusion treatment by the number of exuviae that may have been attracted to this treatment due to the increased structural complexity resulting from the presence of the cage; Table 1). This adjustment conservatively lowered the estimated number of predation events and consequently reduced the effects of the mechanical defense and structural complexity treatments. Because we used aggregate date, we could not use regression‐based models to analyze these data. Alternatively, we used G‐tests to compare treatments.

### 2.4 Sentinel prey experiment

To determine predator–prey interactions directly, rather than relying on the assumptions of the first experiment alone, we performed a second experiment in which we pinned thawed odonate nymphs onto the artificial plants and estimated survival free from the effects of odonate preference on 10, 18, 22, and 24 July, 2014. Nymphs were collected from ponds at our study site and kept in a freezer until use. We used the same artificial plants in the first experiment but removed the cages from the predator‐exclusion treatments allowing predators access to all plants. For each of the four trials of this experiment, one odonate nymph was pinned to the stem of each artificial plant ~30 min before sunrise (~05:00) as we observed that Red‐winged Blackbirds (*Agelaius phoeniceus*), the assumed primary predator at our study site, began foraging roughly at sunrise. After 2 hrs of predator exposure, we returned to the site and recorded whether the pinned nymphs were present or absent. Previous observations showed that it took upwards of an hour for birds to forage in areas we disturbed. Consequently, the effective predation exposure duration was approximately 1 hr.

We used glmm to determine the proportion of nymphs removed from the artificial plants. The function “glmer” was used to fit models with a binomial error distribution and logit link function (Breslow & Clayton, 1993). As before, we employed a stepwise deletion approach. Complexity and mechanical defense were included as fixed effects and “date” and “shore location” were included as random effects. Video analysis revealed spatial autocorrelation of bird foraging (see Section 2.3). To account for this, we included a neighbor predation factor (NPF; i.e., whether or not an individual nymph's neighbor was consumed).

### 2.5 Predator identity and behavior

To confirm that Red‐winged Blackbirds (*A. phoeniceus*) were the predominant predators in this system and that the presence of odonate exuviae is a valid proxy for a successful emergence event, we deployed motion sensor no‐glow black LED video cameras (Bushnell model # 119439) during four 7–8 day periods from 24 June 2014, to 24 July 2014. We deployed eight cameras at two experimental plants for each of the four uncaged complexity and defense treatments for both experiments: (1) Molting preference and predation experiment and (2) Sentinel prey experiment. To account for predation rate biases the presence of video cameras might incur on uncaged treatments (i.e., attracting or detracting predators; Richardson, Gardali, & Jenkins, 2009), we also deployed 8 sham cameras fashioned from wooden blocks of same dimension and color to real cameras at two experimental plants for each of the four exclosure and defense treatments.

## 3 Results

### 3.1 Exuviae surveys

**Figure 3:**
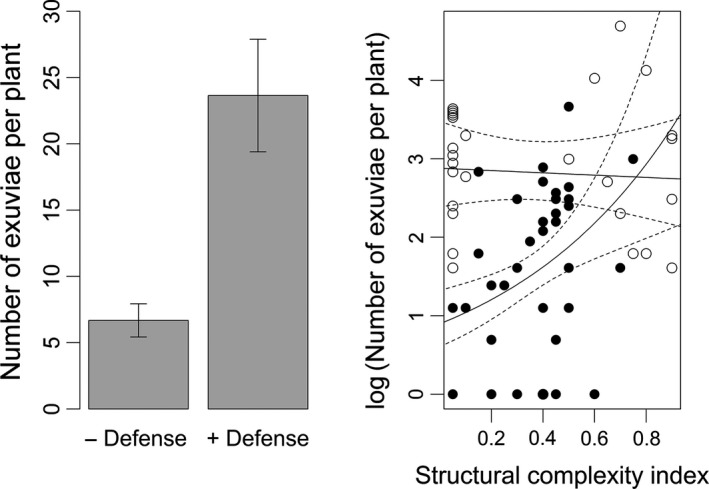
Number of odonate exuviae found within naturally occurring plants surrounding a pond at our study site. The left panel shows the mean ±1 *SE* of exuviae per plant categorized as possessing (+; *n* = 28) or not possessing (−; *n* = 37) a mechanical defense. The right panel illustrates the relationship between the log‐transformed number of exuviae per plant and an index of plant structural complexity. Open and filled circles represent plants with and without mechanical defenses, respectively. Lines represent best‐fit regression and the 95% confidence interval

Plant traits influenced the distribution of dragonfly exuviae on naturally occurring shoreline plants. Of the 65 surveyed plants, 38% possessed mechanical defenses and had 254.9% more exuviae than those that did not (Figure 3a; glmm, *Z* = 3.41, *p* = .0007; see supplemental information). Using our complexity index (see Section 2), structural complexity ranged from 0.05 to 0.90, with a mean and *SD* of 0.36 and 0.27, respectively. Structural complexity was marginally significant (glmm, *Z* = 2.01, *p* = .045) as well as its interaction with mechanical defenses (Figure 3b; glmm, *Z* = −1.98, *p* = .048). For plants without mechanical defenses, an increase of 1 unit of structural complexity resulted in the average addition of 4.4 exuviae (C, plates 3 and 4 in Appendix S2). There was weak correlation between structural complexity and exuviae for plants with mechanical defenses (parameter est ± *SE*; 0.15 ± 0.25 exuviae).

### 3.2 Molting preference and predation experiment

**Figure 4:**
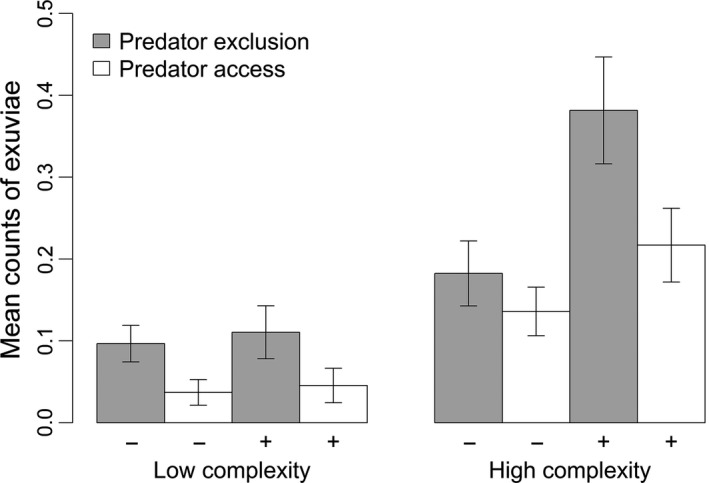
Counts of exuviae (mean ±1 *SE*) in artificial plants with high and low structural complexity and with (+) and without (−) simulated mechanical defenses. Half of the treatments excluded predators but allowed nymphs access, while the half of the treatments allowed both nymphs and predators access for a total of 136 plants equally divided among treatments (*n* = 17 plants per treatment)

We found support for both mechanical defenses and structural complexity reducing predation. In the full model, all three factors, predator access, structural complexity, and mechanical defenses, influenced the number of odonate exuviae found on the artificial plants (Figure 4, Table 2a). On average, the predator‐exclusion treatment had 65% more exuviae than the predator‐access treatment. A predator‐free control experiment showed a weak nymph preference for the cages used in the exclusion treatment (D in Appendix S2). Therefore, the difference between the predator‐exclusion and predator‐access treatments cannot be completely attributed to predation; this difference is also in part due to nymph preferences for cages. Finding an effect of cage restricted our analysis to main effects only thereby preventing us from testing higher order interactive effects involving predator access (i.e., *P* × *C*,* P* × *D*,* P* × *C* × *D*). We instead used corrected aggregate values (see Section 2) to calculate inferred survival. Inferred survival was 62% higher in the high than low structural complexity treatment (*G* = 11.24 *df* = 1, *p* = .0008). Inferred survival was 4% higher in the mechanical defense treatment, but this value was not statistically significant nor was the interaction between these two factors (*p* > .05).

**Table 2:** Results from the molting preference and predation experiment (a) the *combined* model shows the output of a generalized linear mixed effect minimal adequate model for the number of odonate exuviae on artificial plants from a factorial experiment where the additive factors included complexity (high or low), mechanical defense (with [+] or without [−]), and predators (excluded [−] or access [+]). Interactive effects involving predators were not included in the maximal model; (b) the *preference* model used the same modeling approach as in the *combined model* but calculated parameter estimates for the predator‐exclusion treatment only

Fixed effects	Estimate	Std. error	*Z*	*p*‐Value
(a) Combined				
Intercept	−2.548	0.403	−6.32	2.6e‐10
Complexity high	1.205	0.188	6.42	1.4e‐10
Defense+	0.462	0.173	2.67	7.6e‐3
Predator+	−0.642	0.174	−3.68	2.4e‐05
(b) Preference				
Intercept	−2.827	0.391	−7.24	4.5e‐13
Complexity high	1.174	0.188	6.24	4.3e‐10
Defense+	0.479	0.175	2.74	0.0061

Parameter estimates are on a log scale.

We assessed nymph molting site preference by examining the number of exuviae in the predator‐exclusion treatment: Artificial plants with high structural complexity had 175% more exuviae than those with low structural complexity; artificial plants with mechanical defenses had 77% more exuviae than those without (Figure 4, Table 2b). The mechanical defense and structural complexity interaction (i.e., *D* × *C*) was not significant (*p* < .05). Detecting a preference for mechanical defenses corroborates findings from a pilot experiment where actual thistle stems (*C. vulgare*) were used to test preference; in the absence of predators, 105% more exuviae were on control thistle stems compared to thistle stems that had their prickles removed (E in Appendix S2).

### 3.3 Sentinel prey experiment

**Figure 5:**
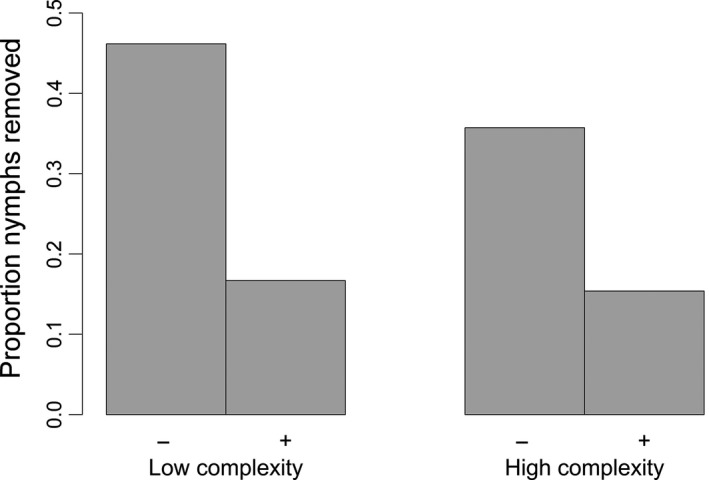
The proportion of pinned odonate nymphs removed from artificial plants with high and low structural complexity and with (+) and without (−) simulated mechanical defenses during the first trial (i.e., naïve population). A total of 52 plants divided among the four treatments were used, with one nymph affixed to each plant

The presence of mechanical defenses but not structural complexity influenced the proportion of nymphs removed by birds (Figure 5, Table 3). We did not detect a significant interaction between structural complexity and mechanical defenses (*p* > .05). However, there was a significant interaction between mechanical defenses and the neighbor predation factor (NPF).

**Table 3:** Results of generalized linear mixed effect minimal adequate models for the number of odonate pinned nymphs removed from artificial plants with high and low structural complexity and with (+) and without (−) simulated mechanical defenses

Fixed effects	Estimate	Std. error	*Z*	*p*‐Value
Intercept	−1.686	0.344	−4.899	9.65e‐07
Defense+	−1.748	0.797	−2.194	0.028
NPF+	2.012	0.501	4.016	5.93e‐05
Defense+ × NPF+	2.221	0.963	2.305	0.021

To control for predator conditioning and its effect on foraging behavior, a neighbor predation factor (NPF; neighbor consumed [+], neighbor not consumed [−]) was used. Structural complexity did not increase model fit according to a likelihood ratio test and consequently was not included in the minimal adequate models.

Parameter estimates are on a logit scale.

### 3.4 Predator identity and behavior

Experimental emergence preference plants and pinned nymphs were exposed to video cameras for a total of 4,053 camera hours. Upon analysis, we found that the motion‐activated sensor which triggered recording was often too slow to capture predation events. Consequently, unlike the molting preference and predation experiment and sentinel prey experiment, the video analysis cannot be used to quantify predation pressure. Despite this technical shortcoming, we recorded six predation events (4 from the molting preference and predation experiment and 2 from the sentinel prey experiment); all were by Red‐winged Blackbirds (F and G in Appendix S2). In all cases, the bird picked up the nymph pre‐ or during emergence, leaving no exuviae behind. This finding provides support for the assumption that the presence of exuviae is a valid proxy for successful molting events.

## 4 Discussion

We experimentally tested the relative importance of two functional plant traits in a simple predator–prey relationship based on an observational study, where more odonate exuviae were associated with naturally occurring plants that possessed mechanical defenses and a structurally complex architecture compared to those that did not possess mechanical defenses and were less structurally complex. Odonates preferred to emerge on *artificial* plants with high structural complexity over those with low structural complexity and, to a lesser extent, they preferred artificial plants with mechanical defenses over those lacking these defenses. The presence of both high structural complexity (simulated whorls of leaves) and mechanical defenses (simulated spines) decreased nymph predation rates by Red‐winged Blackbirds. Consequently, our experiments and observational study suggest that through these functional traits, plants can facilitate emerging odonates by providing an associational refuge from avian predators. Until recently, “complexity” studies have lumped elements contributing to structural complexity together (Kovalenko et al., 2012). In a pioneering study by Beck (2000), different elements contributing to the heterogeneity of large‐scale habitat structure (e.g., rocks, trees, pits, and pneumatophores) were manipulated to understand their specific influence on intertidal gastropod densities. Working at the microhabitat scale of a small herbaceous plant, we separated mechanical defenses from structural complexity and quantified their relative importance as well as interactive effects on odonate nymph preference and survival. Though mechanical defenses are structural components, we posit that the mechanism by which they disrupt predator–prey dynamics is different than that of structural complexity. The mechanical defenses on our artificial plants (i.e., sewing pins) are too small relative to the body size of nymphs to prevent visual detection from avian predators. Similarly, the interstitial space created by the pins was not sufficiently small relative to predator body size to prevent access by that predator. Video observations showed that birds could physically reach pinned nymphs in all treatments but did so less often when plants were physically defended as evidenced by the sentinel prey experiment (G in Appendix S2). Based on these findings, it is likely that Red‐winged Blackbirds preferred to remove nymphs from undefended plants to avoid injury and not because of lack of visual detection or access.

### 4.1 Structural complexity

Consistent with the well‐supported hypothesis that increased structural complexity decreases predation effects, we found that high structural complexity increased nymph survival in the molting preference and predation experiment (Figure 4, Table 2a). However, high structural complexity did not confer a survival advantage in the sentinel prey experiment (Figure 5, Table 3). One likely explanation for this is that in our sentinel prey experiment, we pinned nymphs to the stems of all artificial plants on the pond side. However, on artificial and natural occurring plants, when allowed to choose, nymphs chose a variety of emergence sites, such as on the underside of the artificial leaves (C in Appendix S2). Video analysis demonstrated that Red‐winged Blackbirds visually detected pinned nymphs from the ground while walking between plants. As a result, the pinned nymphs were likely easily detectable and accessible. Red‐winged Blackbird prey searching behavior is rapidly influenced by previous experiences with prey (Alcock, 1973). Consequently, predictably placing nymphs on the stems of artificial plants and not in more concealed locations likely decreased the effect of structural complexity in reducing prey detection. This does not reflect an inadequacy in the artificial plant design, but rather not using them in a manner that adequately simulated site selection of emerging individuals. However, including multiple nymph pinning sites (e.g., under leaves) on the artificial plants was logistically infeasible. The lack of supporting evidence from the sentinel prey experiment for the influence of structural complexity on odonate survival found in the preference and survival experiment likely understates its true importance. However, detecting an effect of structural complexity in the molting preference and predation experiment, where some nymphs were likely more concealed compared to the sentinel prey experiment, supports that established mechanism of structural complexity: reduction in prey detection by predators.

During the first trial of the sentinel prey experiment, 155% more nymphs suffered predation on plants which lacked mechanical defenses compared to those that had mechanical defenses (Figure 5). In subsequent trials (total trials, *n* = 4), the effect of mechanical defenses, though significant, was reduced (Table 3). This reduction in the effect size is likely due to birds associating our artificial plants with food rewards, eventually leading to spatial autocorrelation of foraging with each successive trial (i.e., predator conditioning; Ehlinger, 1989; Jones, Castellanos, & Weiss, 2002; Savino & Stein, 1982; Werner, Mittelbach, & Hall, 1981). The intensity of spatial autocorrelation is likely an artifact of our experimental design. Artificial plants were placed in a line, 30 cm apart from one another. The significant interaction between mechanical defenses and the neighbor predation factor (i.e., D X NFP; Table 3) in combination with several recorded predation events (G in Appendix S2) revealed that Red‐winged Blackbirds first removed nymphs from artificial plants without mechanical defenses; they then moved down the row of plants removing nymphs regardless of treatment, seemingly because the birds were aware that each successive plant would have food. The density of molting odonates on any given day is lower than that of naturally occurring plants along the shore of our focal pond. Consequently, the location of exuviae in an unmanipulated system is not as easily predictable and would likely require greater foraging effort by Red‐winged Blackbirds.

Predator conditioning may also explain why the high complexity, with defenses treatment experienced higher predation than the high complexity, without defenses treatment (Figure 4). Birds may have targeted artificial plants with the highest aggregation of emerging nymphs (or exuviae), a result consistent with other studies which demonstrated preferential foraging on individual plants associated with the highest abundances of prey (Flower, Long, Knight, & Rebbeck, 2014; Heinrich & Collins, 1983).

Odonate nymphs showed a strong preference for artificial plants with high structural complexity in the molting preference and predation experiment (Figure 4, Table 2b). This finding was corroborated by the predator‐free, control experiment, where nymphs were more likely to molt on artificial plants in the high complexity treatment as well as in the low structural complexity treatment if a predator‐exclusion cage was present—nymphs likely perceived the exclusion cage as additional structural complexity (D in Appendix S2). There are several reasons that might explain this preference for high structural complexity. First, emerging nymphs might seek out complex substrate due to the potential protection it may afford. Dragonfly nymphs as well as many other invertebrates have been shown to actively seek refuge in submerged aquatic vegetation with complex structure (Grutters et al., 2015; Lauridsen & Lodge, 1996). A second explanation is that structurally complex plants are simply easier to detect by dragonflies, which orient visually (Corbet & Brooks, 2008) and as a consequence, unintentionally benefit from the superior refuge provisioning of structurally complex plants. A third explanation is that this finding is a result of the often confounded nature between surface area and structural complexity (Loke & Todd, 2016). If the trajectory of molting nymphs is completely random, then it is expected that more exuviae would be found on structurally complex plants; exuviae should positively correlate with complexity simply due to the increased surface area in which nymphs may randomly encounter. We controlled for this issue by keeping the stem surface area, the part of our artificial plants crawling nymphs encountered, equal across all treatments. The difference in surface area between treatments was a function of simulated leaves and the predator‐exclusion cages, which were well beyond the reach of nymphs. Video analysis showed that once a nymph chose an artificial plant and moved upward, it remained in that plant until it flew away as an adult or was removed by Red‐winged Blackbirds. Constructing taller and/or wider artificial plants in the low structural complexity treatment to keep surface to keep surface area constant between treatments could have also influenced nymph preference, and thus it was decided against and post hoc analysis are generally considered inadequate or inappropriate (Loke & Todd, 2016; Whittingham, Stephens, Bradbury, & Freckleton, 2006). The influence of structural complexity on nymph survival, however, as was determined in the sentinel prey experiment, does not suffer from these potential issues as we controlled the density of pinned nymphs on each plant.

### 4.2 Mechanical defenses

In addition to structural complexity, nymphs preferred artificial plants with mechanical defenses (Figure 4, Table 2b). This finding is consistent with a pilot study where nymphs were found to prefer thistles whose prickles were not removed compared to those that were (F in Appendix S2). Unlike the high structural complexity treatment that is highly conspicuous and consequently may provide a visual cue for premolting nymphs, the simulated mechanical defenses are fairly small and likely difficult to see from a distance (i.e., from the focal pond). However, less is known about nymph vision compared to the adult stage (but see Seki, Fujishita, & Obana, 1989), which is thought to have the most advanced eyes within the class Insecta (Bybee, Johnson, Gering, Whiting, & Crandall, 2012).

The sentinel prey experiment demonstrated that artificial plants with mechanical defenses provided a predation refuge for pinned nymphs (Figure 5, Table 3). Though likely common in nature, only a few studies have rigorously tested the exploitation of mechanical defenses as the underlying mechanism affording protection via associational refuge. As with structural complexity, most of these studies were conducted in marine systems (e.g., Bittick, Nicholas, Peterson, & Stewart, 2010; Stier et al., 2008; Townsend & Bologna, 2007). In a terrestrial example, Grof‐Tisza et al. (2015) found that an Erebid caterpillar left its host plant to pupate in mechanically defended plants (also thistles). This niche shift greatly reduced predation by rodent predators during the relatively vulnerable pupal stage. Despite the paucity of experimental evidence for the exploitation of mechanical defenses in terrestrial systems, there are numerous natural history observations that support this mechanism of refuge provisioning, including nest building in cacti by the cactus ferruginous pygmy owl (*Glaucidium brasilianum cactorum* and Gila woodpecker (*Melanerpes uropygialis*; Flesch, 1999) and perch selection by the piny‐tailed Iguana (*Ctenosaura hemilopha*; Blázquez & RodrÍguez‐Estrella, 1997). It is likely that the benefits received by associating organisms vary substantially depending on the type, size, and density of mechanical defenses on habitat‐forming species. However, this study and those cited herein illustrate the potential importance mechanical defenses may play in dictating refuge space and subsequently trophic structure.

Taken together, the molting preference and survival and sentinel prey experiments help explain patterns in the observational study (Figure 3). Naturally occurring plants with high structural complexity and mechanical defenses had the highest numbers of exuviae. This is likely driven by both preference for structural complexity (i.e., leaves and simulated prickles) and increased nymph survival due to the presence of mechanical defenses. Structural complexity may also be an important factor increasing nymph survival, but this mechanism was not supported by our experiments. Accordingly, plants with low structural complexity and no mechanical defenses had the lowest numbers of exuviae. Interestingly, increasing structural complexity for mechanically defended plants did not correlate with exuviae number. This result is largely driven by star thistle (*C. solstitialis*). Star thistle is a tall, spindly plant with sparse foliage and mechanically defended flowers concentrated at stem terminals. This architecture leads to a low structurally complex interior surrounded by a shield of long spines. Previous work demonstrated that these spines deter herbivores (Callihan, Lass, Hunt, & Pritchard, 1995) as well some flying insects (Agrawal et al., 2000), lending support to the hypothesis that these defenses are also effective against birds. Unlike more structurally complex plants, where exuviae were found throughout the plant—generally on the underside of leaves—exuviae in star thistle were concentrated in the center of the plant (C in Appendix S2). Though not addressed in this study, this finding suggests that the location of mechanical defenses may be as important as their presence, depending on plant architecture. Detecting an effect of plant traits with our rather simple artificial plants suggests that the structural complexity and mechanical defenses of real plants do facilitate associating species, especially considering that naturally occurring plants can be larger, more complex, and more heavily defended in some system.

Bull thistle (*C. vulgare*) and star thistle (*C. solstitialis*), which are considered invasive in the United States, were the only species to possess ridged, spinose mechanical defenses within our survey plots. In light of our findings regarding mechanical defenses, these non‐native species may positively affect native odonates through the provisioning of an associational predation refuge. This finding is consistent with other studies which demonstrated that novel habitat created by non‐native species can positively facilitate native species (Bittick et al., 2010; Schwindt, Bortolus, & Iribarne, 2001; Valinoti et al., 2011).

## 5 Conclusion

Functional plant traits, specifically structural complexity and mechanical defenses, influenced the preference and survival of emerging odonates. Structural complexity had a strong effect on nymph preference and in agreement with our expectations, conferred a survival advantage on our artificial plants. Mechanical defenses affected preference but with a weaker magnitude than structural complexity and also increased nymph survival. We also detected a preference for mechanical defenses in a pilot study that used actual thistle plants (E in Appendix S2). Our experiments in conjunction with video analysis suggest that the mechanistic underpinning of mechanical defenses is different than that of structural complexity: Mechanical defenses prevented access of predators to prey likely due to injury avoidance; structural complexity (in terms of the density of particular structural elements) likely decreased prey detection rates. We suggest that future studies attempt to separate the importance of elements contributing to overall structural complexity to elucidate mechanisms underlying associational refuge provisioning.

## Conflict of Interest

None declared.

## Data Accessibility

We intend to make our data available through Dryad.
