# Supplementary material for: Plant structural complexity and mechanical defenses mediate predator–prey interactions in an odonate–bird system
Source: Ecol Evol. 2017 Feb 10;7(5):1650–9. doi: 10.1002/ece3.2705 (PMC5330893; doi:10.1002/ece3.2705)
Supplement: Supplementary file 2 [file ECE3-7-1650-s002.docx]

**Appendices**

**Appendix A:** Structural complexity schematic diagram

**Appendix B**: Methods for simulating biological relevant mechanical defenses on artificial plants

**Appendix C**. Plates of artificial plants around the focal pond and exuviae in mechanically defended plants

**Appendix D**. Exclusion treatment control experiment

**Appendix E.** Preference experiment with thistles

**Appendix F**. Plate of Red-winged Blackbird with nymphs

**Appendix G.** Plate of Red-winged Blackbird entering canopy of artificial plant to consume a tethered nymph

**Appendix A**

Structural complexity conceptual schematic diagram.


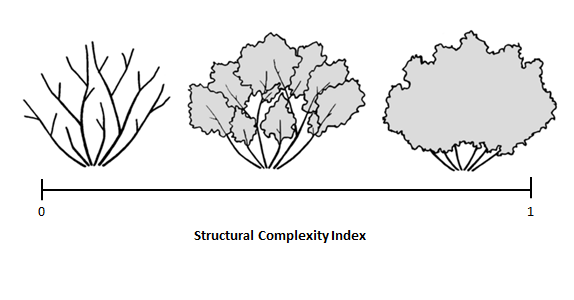


Illustration by Jane G. Smith

**Appendix B**

*Mechanical defense treatment simulation*

To simulate mechanical defenses, we pushed sewing pins through both the stems and leaves of our artificial plants. To ensure biological relevance, we used the same pin density and length as the prickle density and length of the Italian thistle (*Carduus pycnocephalus)* found at our study site (mean ± SD: 0.58±0.08 prickles per cm, 10.2±0.67 mm in length).

**Appendix C**

**
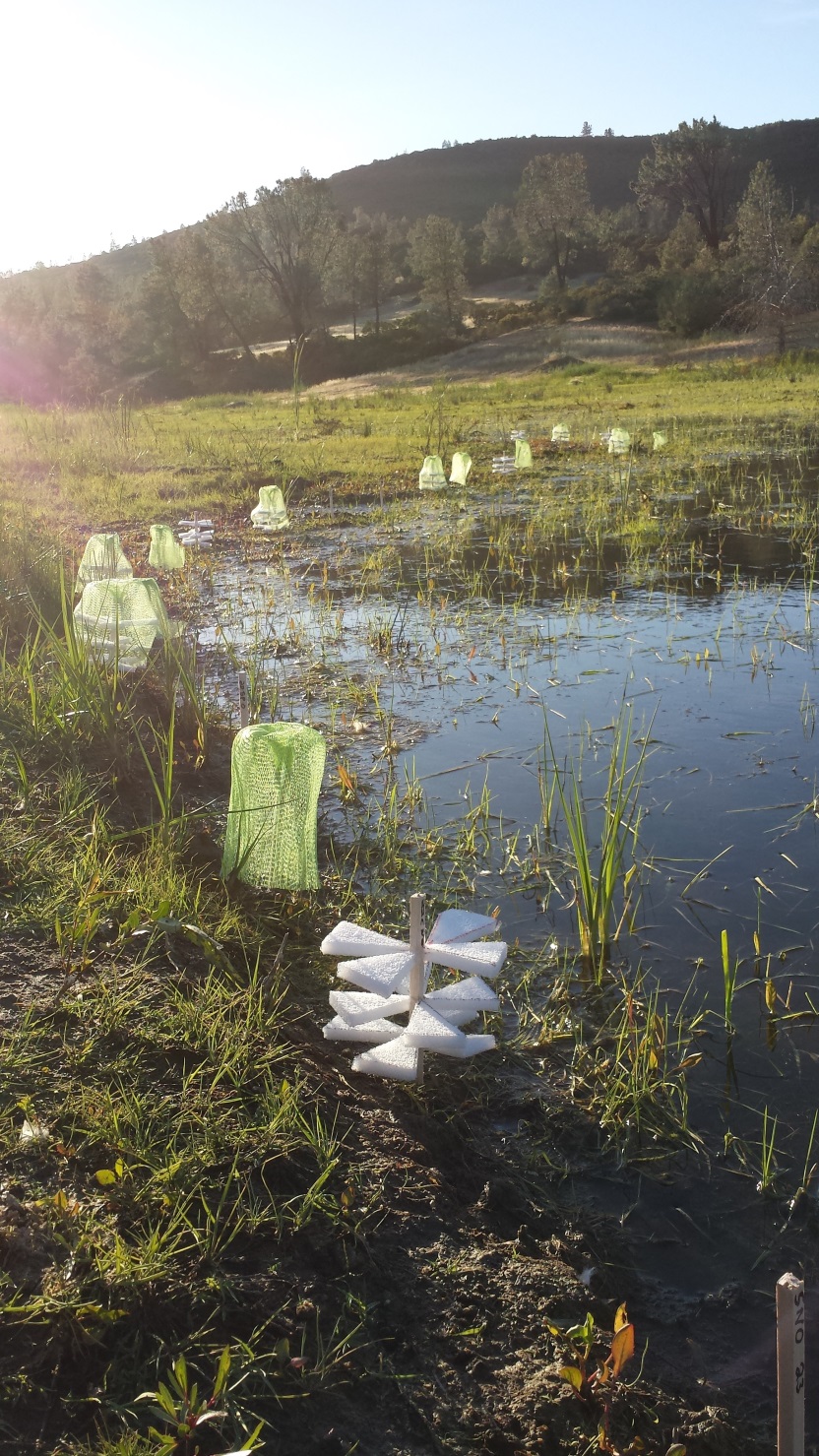
**

Plate 1. Artificial plants surrounding the focal pond. Photo credit: Patrick Grof-Tisza


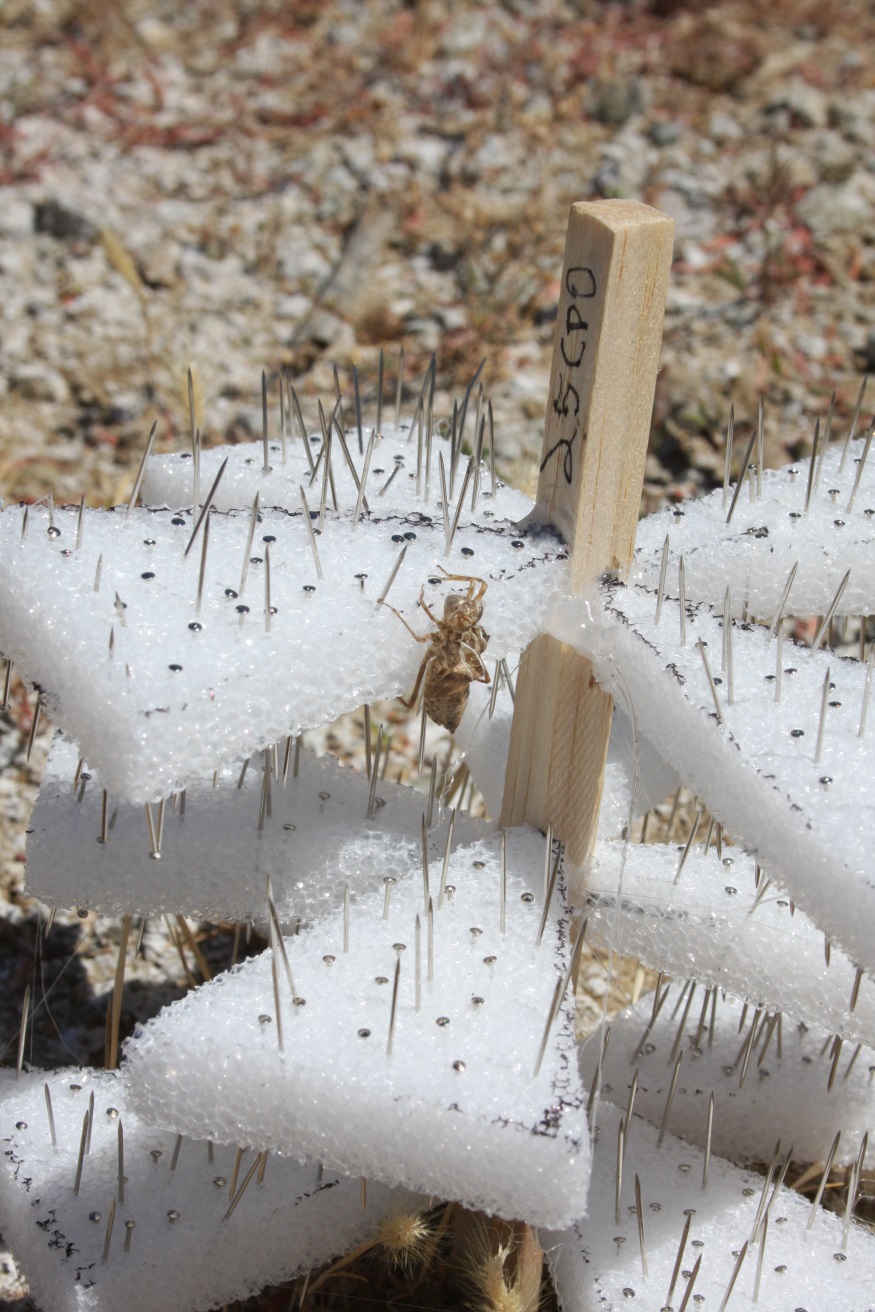


Plate 2. *Sympetrum corruptum* exuviae on artificial plant with high structural complexity and mechanical defenses. Photo credit: Patrick Grof-Tisza

**
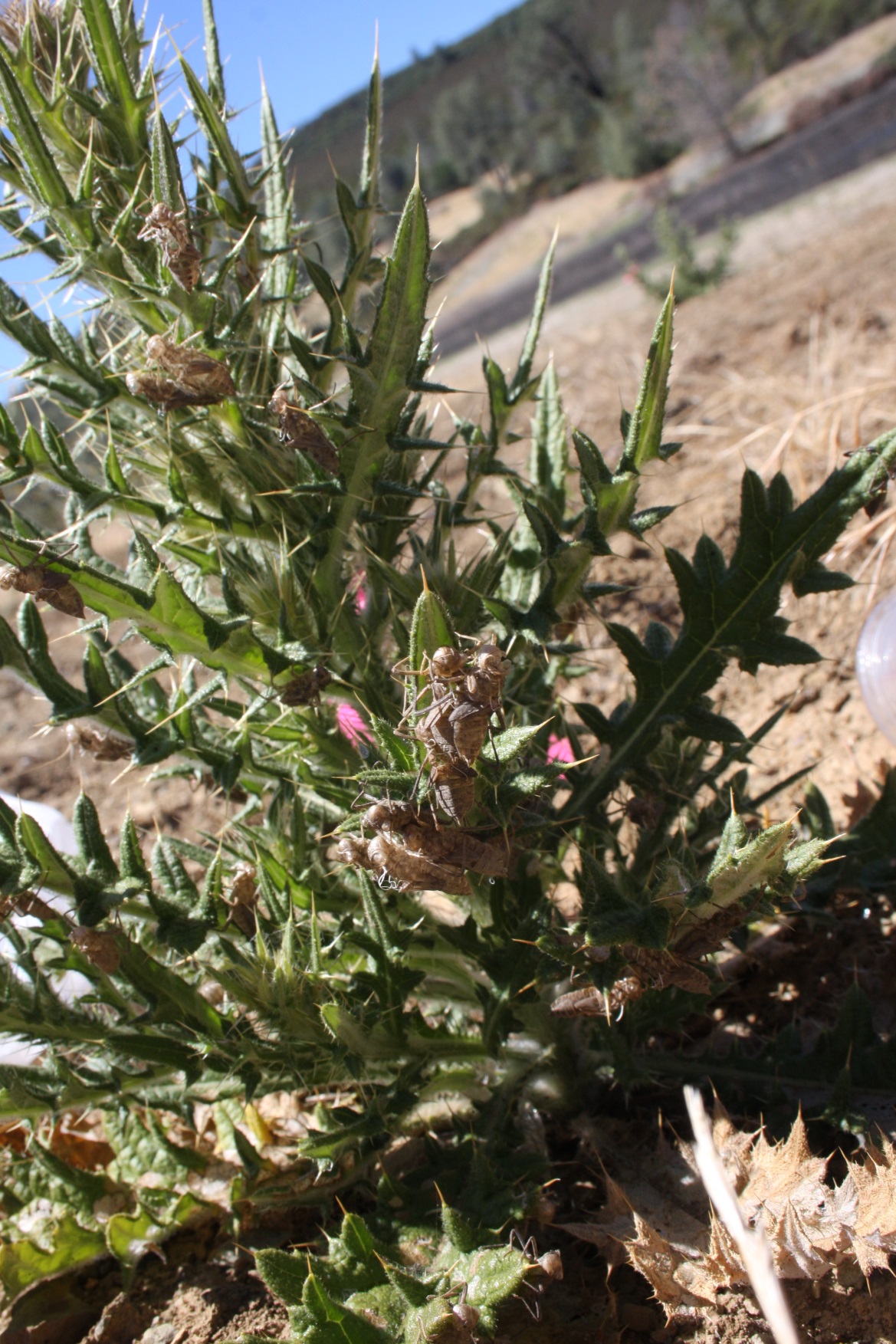
**

Plate 3. Dragonfly exuviae on bull thistle (*Cirsium vlugare*) at our study site. Photo Credit: Eric LoPresti

**
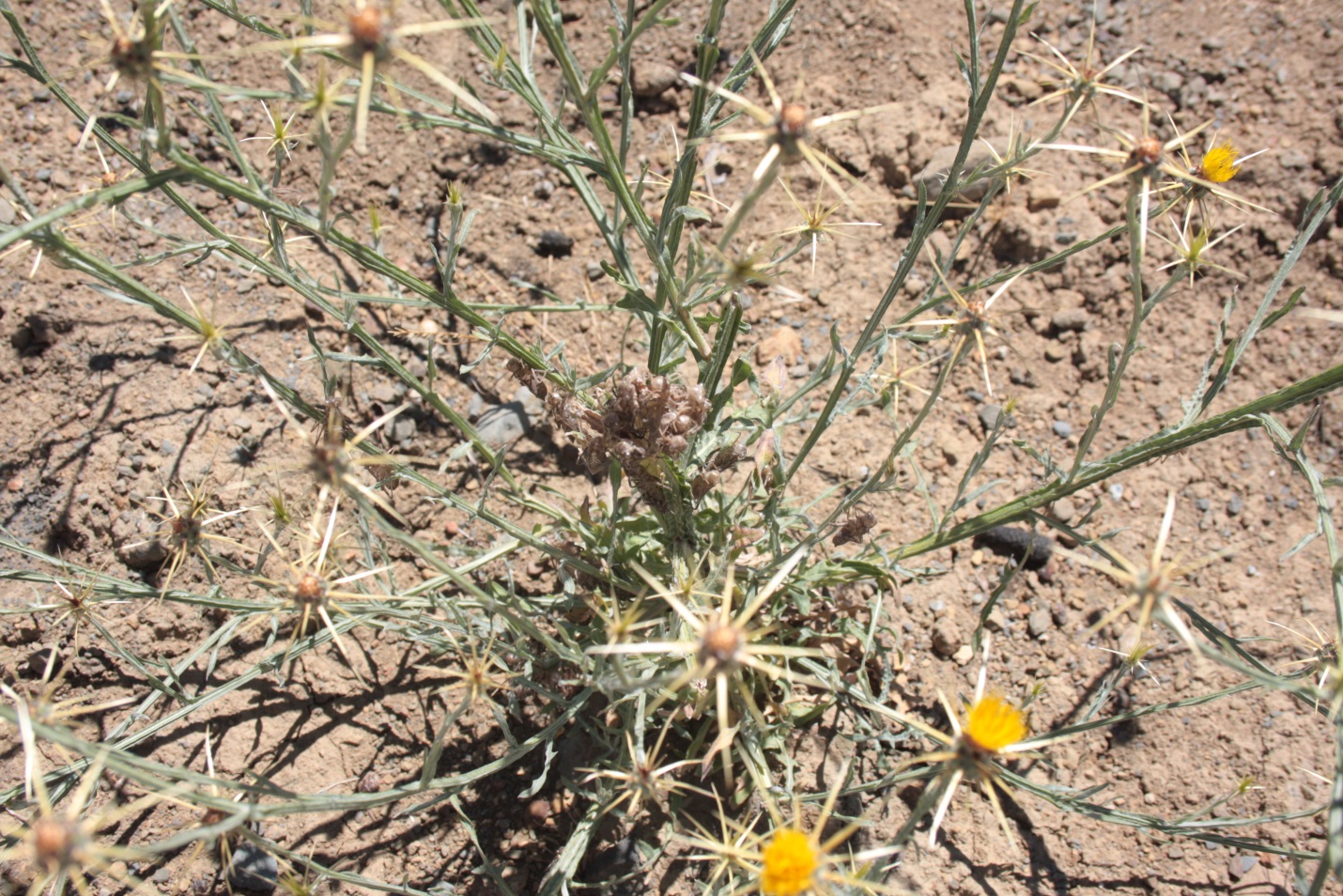
**

Plate 4. Dragonfly exuviae (clustered in center) on star thistle (*Centaurea solstitialis*) at our study site. Photo credit: Eric LoPresti

**
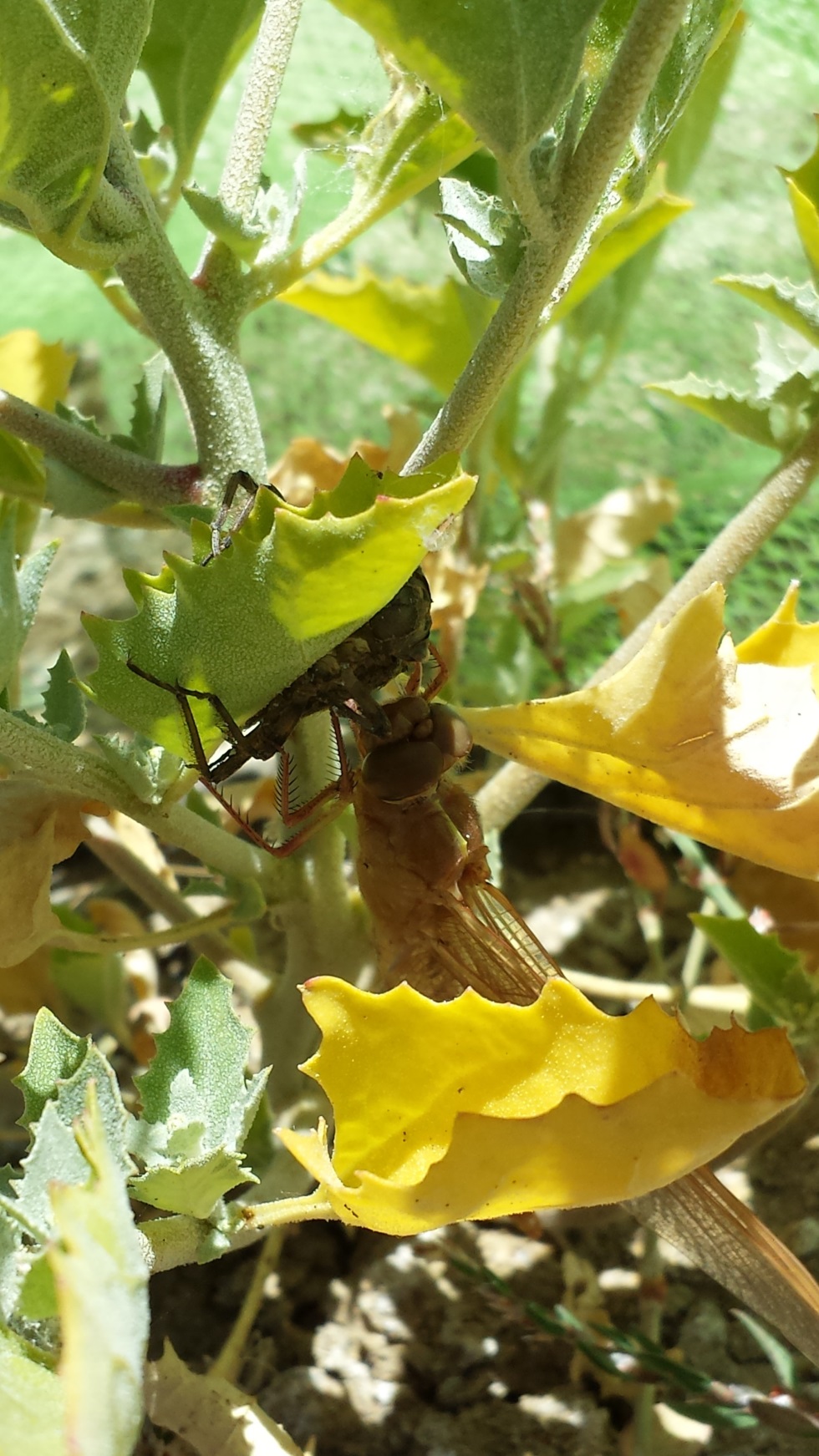
**

Plate 5. Molting dragonfly emerging from its exuviae on the underside of an *Atriplex rosea* leaf. Photo credit: Patrick Grof-Tisza

**Appendix D**

*Exclusion treatment control experiment*

To determine the effect of the nets suspended over artificial plants used to exclude birds in the field experiment, we conducted a predator-free control experiment. We deployed all plant treatment combinations in two 200-gallon cattle tanks covered with netting to prevent birds at UC Davis’ Center for Aquatic Biology and Aquaculture, Davis, California (plate). Cattle tanks were filled and left uncovered for 12 months allowing natural odonate recruitment. The common species in this experiment were *Enallagma* spp., *Ischnura* spp., *Tramea lacerata, Anax junius, Pantala hymanea* and *P. flavescens*, all of which are common at the focal pond as well. Artificial plants (n=16) were checked weekly and the number of exuviae were removed and counted from 24 August to 27 September 2014.

To determine the importance of the exclusion treatment as well as mechanical defenses and structural complexity, we used generalized linear mixed models (glmm). The function ‘glmmadmb’ was used to fit models in R (3.0.2) with a negative binomial error distribution and log link function. Using a stepwise deletion approach from the maximal model, including interactions, the minimal adequate model was selected. The minimal model consisted of only significant terms assessed by residual deviances to a chi-square distribution with residual degrees of freedom (Crawley 2007). Cattle tank identity and date were included as random effects in the model. Based on visual analysis (see figure), we conducted a post-hoc analysis similar to what is described above to determine the effect of cage in the low complexity treatment.

Structural complexity was the only factor found to be important in the full model (Fig. Table). However, there was a weak effect of cage when limiting the data to the low complexity treatment only (*Χ ^2^* = 3.96, P= 0.047). Consequently, there is some evidence to suggest that the presence of the suspended net cage over the artificial plants in the exclusion treatment may have influence odonate behavior at least in the low complexity treatment.

**
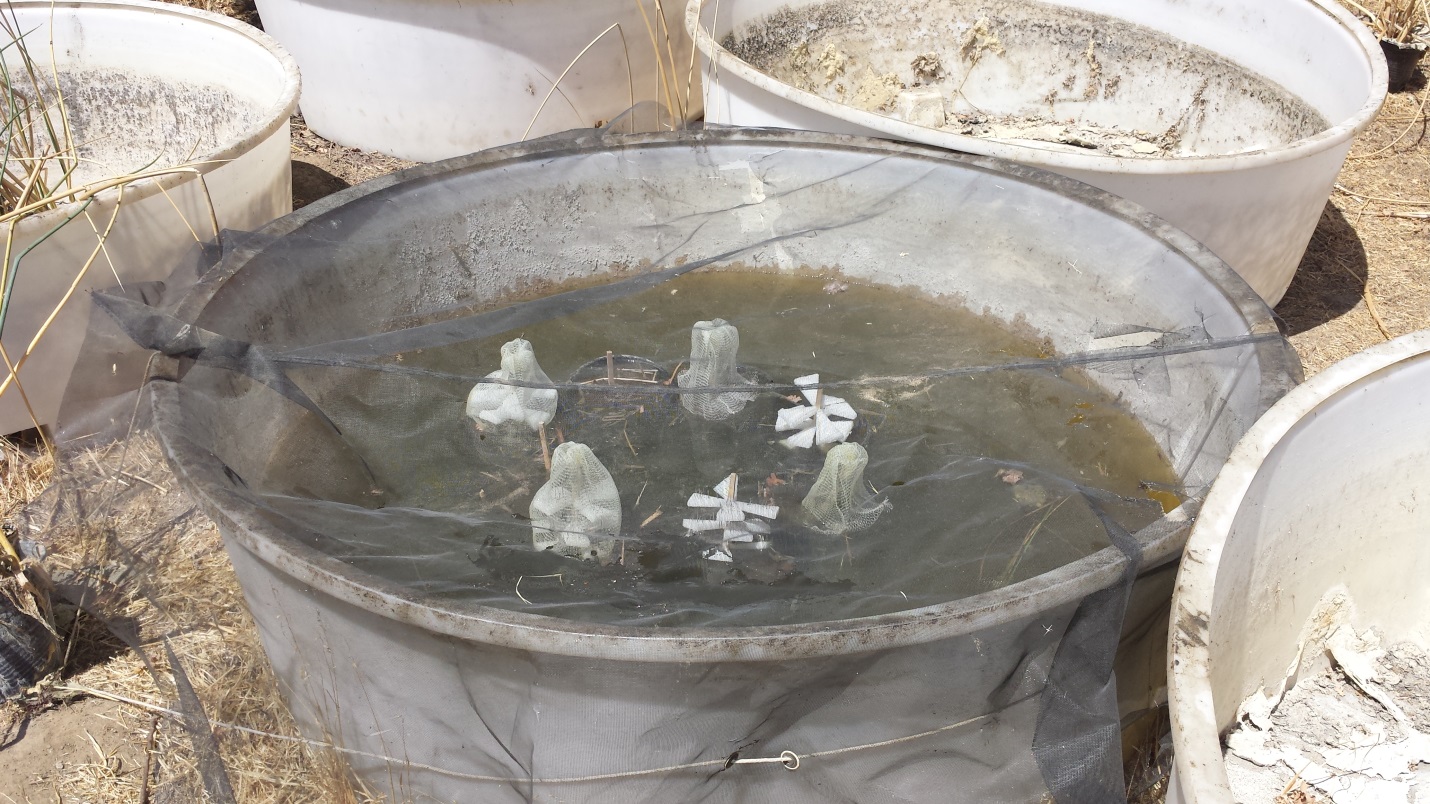
**

Plate. All treatment combinations of artificial plants in a cattle tank covered with netting to prevent access by avian predators.


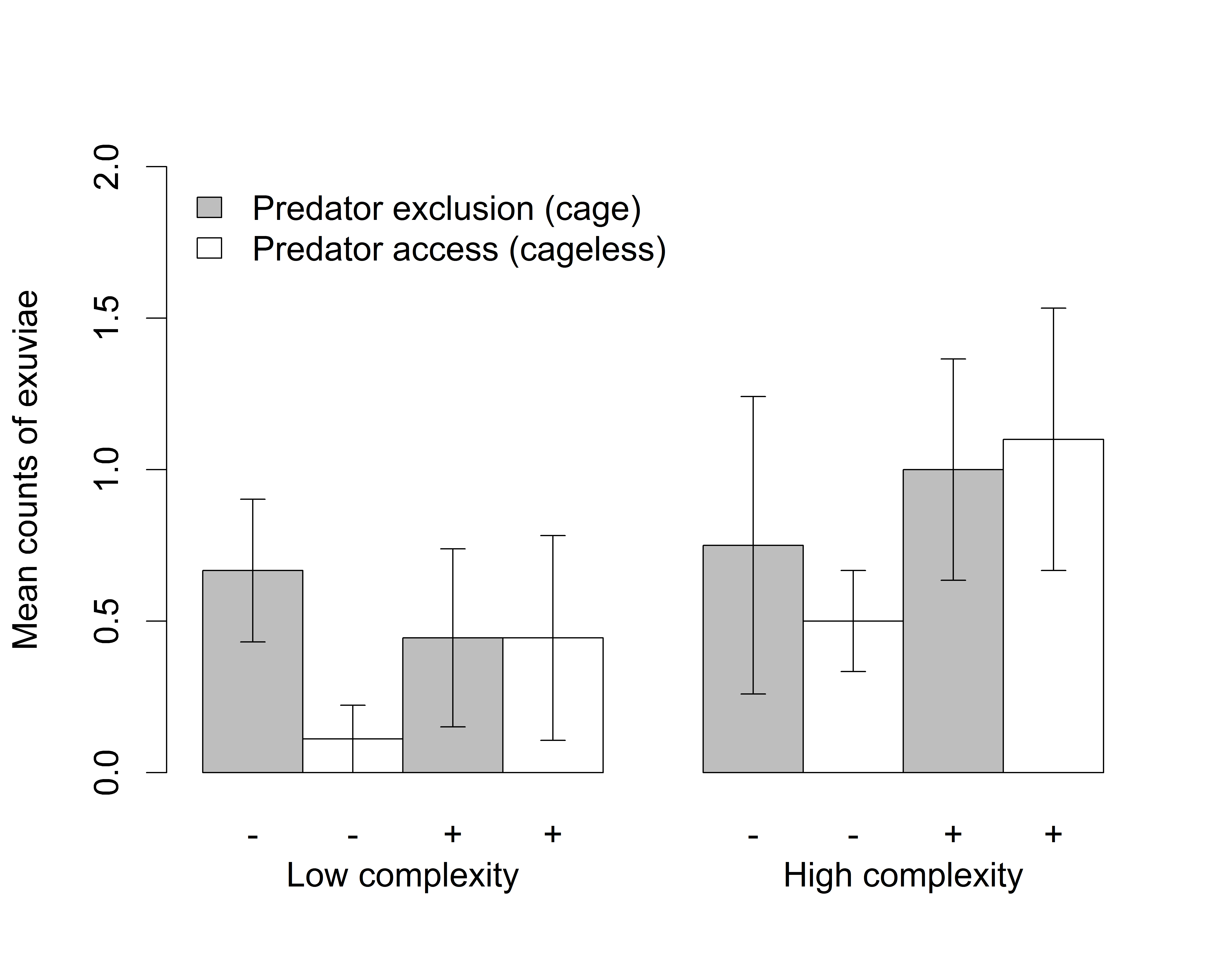


Figure. Counts of exuviae (mean ± 1 SE) in structurally simple and complex artificial plants with (+) and without (-) simulated mechanical defenses in predator control experiment. All artificial plants (n=16) were placed in one of two, 200 gallon cattle tanks. Tanks were covered with netting to prevent access by avian predators. Note that, the legend was made to be consistent with the figure from the field study (see manuscript) but is somewhat misleading in this context because predators had access no treatments within this experiment, the predator exclusion cages were within the overall predator exclosure atop the tank.

Table 1. Results of generalized linear mixed effect minimal adequate model for the number of odonate exuviae on artificial plants from the predator – free control factorial experiment. The maximal model included complexity (high [H] or low [L]), mechanical defense (with [+] or without [-]), and predators (excluded (cage) or included (cageless) as fixed effects and date and cattle tank as random effects.

| **Fixed effects** | **Estimate** | **Std. Error** | **Z** | **P-value** |
| --- | --- | --- | --- | --- |
| Intercept | -0.396 | 0.439 | -0.9 | 0.366 |
| Complexity L | -0.718 | 0.341 | -2.1 | 0.036 |

*Parameter estimates are on a log scale*

**Appendix E**

*Nymph preference for mechanical defenses in thistle plants*

To test nymph preference for mechanical defenses, we conducted a choice experiment in cattle tanks using thistles (*Cirsium vulgare*). We collected 30 *C. vulgare* stems from the field and stripped them of their leaves. We removed all prickles from half of the thistle stems with a scalpel while the other half were left unmanipulated. Thistle stems were potted in 15 cm flower pots and attached to plastic garden stakes. We deployed all plants in five 200-gallon cattle tanks at UC Davis’ Center for Aquatic Biology and Aquaculture, Davis, California as described in the *Exclusion treatment control experiment*. Exuviae were removed counted on thistles weekly between 25 July and 12 September 2013.

To determine the importance of the mechanical defenses on preference we used generalized linear mixed models (glmm) as described in the *Exclusion treatment control experiment.*

We found that mechanical defenses (i.e., prickles) affected preference. Thistles that had their mechanical defenses intact had 105% more exuviae than those that had their mechanical defenses removed (table).

Table: Results of generalized linear mixed effect minimal adequate model for the number of odonate exuviae on thistle plants with or without mechanical defenses using date and cattle tank as random effects.

| **Fixed Effect** | **Estimate** | **95% C.I.** | **Z** | **P-Value** |
| --- | --- | --- | --- | --- |
| Intercept | -1.979 | 0.620 | -3.19 | 0.0014 |
| Defenses + | 0.729 | 0.729 | 2.18 | 0.0295 |

*Parameter estimates are on a log scale*

**Appendix F**

*
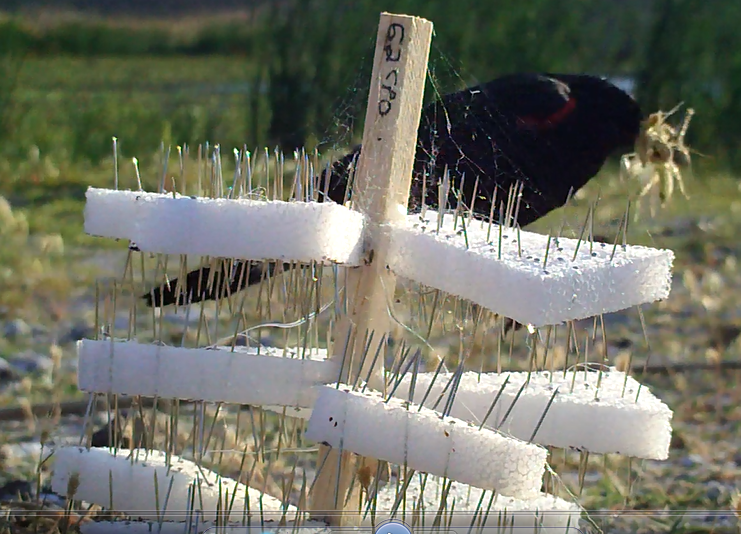
*

Footage from a motion sensor video camera of a Red-winged Blackbird with at least 3 dragonfly nymphs in its bill near an artificial plant with high structural complexity and mechanical defenses, from which one of these nymphs were removed.

**Appendix G**

*
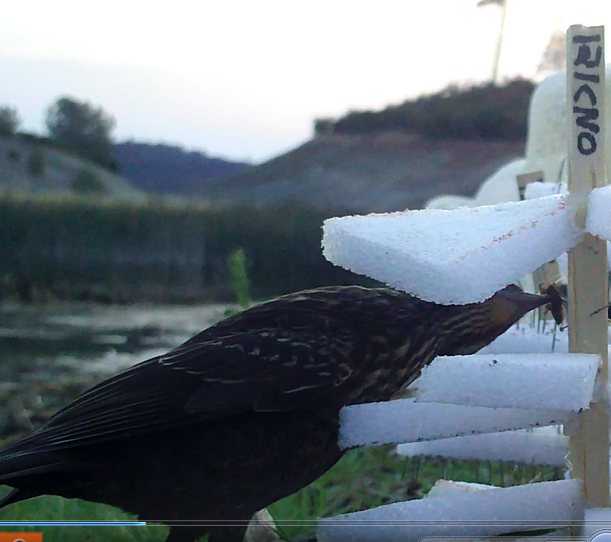
*

Footage from a motion sensor video camera of a Red-winged Blackbird removing a tethered nymph from an artificial plant with high structural complexity and no mechanical defenses.
